# Establishing a national influenza sentinel surveillance system in a limited resource setting, experience of Sierra Leone

**DOI:** 10.1186/1478-4505-11-22

**Published:** 2013-06-24

**Authors:** Senait Kebede, Ishata N Conteh, Christoph A Steffen, Katelijn Vandemaele, Isata Wurie, Wondimagegnehu Alemu, Fredson Kuti-George, Foday Dafae, Amara Jambai, Ali Ahmed Yahaya, Francis Chisaka Kasolo

**Affiliations:** 1 Independent Consultant, 1163 Hidden Spirit Trail, Lawrenceville, GA 30045, USA; 2 WHO Country Office, 21A&B Riverside Drive, P.O. Box 529, Freetown, Sierra Leone; 3 Agence de Médecine Préventive (AMP), Paris, France; 4 Global Influenza Program, World Health Organization, Geneva, Switzerland; 5 Central Public Health Reference Laboratory (CPHRL), Freetown, Sierra Leone; 6 Ministry of Health and Sanitation, 4th Floor Youyi Building, Freetown, Sierra Leone; 7 Disease Prevention and Control Cluster, Disease Surveillance and Response Programme Area, World Health Organization Regional Office for Africa, BP06, Cite Du Djoue, Brazzaville, Congo

## Abstract

**Background:**

Acute respiratory infections remain a leading cause of morbidity and mortality in Sierra Leone; however, similar to other African countries, little is known regarding the contribution of influenza. Routine influenza surveillance is thus a key element to improve understanding of the burden of acute respiratory infections in Africa. In 2011, the World Health Organization (WHO) funded the Strengthening Influenza Sentinel Surveillance in Africa (SISA) project with the goal of developing and strengthening influenza surveillance in eight countries in sub-Saharan Africa, including Sierra Leone. This paper describes the process of establishing a functional Influenza Sentinel Surveillance (ISS) system in Sierra Leone, a post-conflict resource-poor country previously lacking an influenza monitoring system.

**Methods:**

Sierra Leone utilized a systematic approach, including situational assessment, selection of sentinel sites, preparation of implementation plan, adaptation of the standard operating procedures, supervision and training of staff, and monitoring of influenza surveillance activities. The methods used in Sierra Leone were adapted to its specific context, using the Integrated Disease Surveillance and Response (IDSR) strategy as a platform for establishing ISS.

**Results:**

The ISS system started functioning in August 2011 with subsequent capacity to contribute surveillance activity data to global influenza databases, FluID and FluNet, demonstrating a functional influenza surveillance system in Sierra Leone within the period of the WHO SISA project support. Several factors were necessary for successful implementation, including a systematic approach, national ownership, appropriate timing and external support.

**Conclusions:**

The WHO SISA project demonstrated the feasibility of building a functional influenza surveillance system in Sierra Leone, integrated into existing national IDSR system. The ISS system, if sustained long-term, would provide valuable data to determine epidemiological and virological patterns and seasonal trends to assess the influenza disease burden that will ultimately guide national control strategies.

## Background

Influenza remains an important global public health concern. Annual influenza epidemics are estimated to account for 3 to 5 million severe illnesses and 250,000 to 500,000 deaths worldwide [1]. Though the burden of influenza is well understood in temperate regions, mainly in the United States and Europe [2-5], data from tropical regions, including Africa, remains insufficiently understood [6]. This was most apparent during the 2009 pandemic, which demonstrated the need for a strong worldwide influenza surveillance network.

In 2011, the World Health Organization (WHO) funded the Strengthening Influenza Sentinel Surveillance in Africa (SISA) project in which eight countries were selected to receive support to implement or strengthen influenza sentinel surveillance (ISS) and improve epidemiological and virological data collection and reporting through the global databases FluID and FluNet [7,8]. The project was implemented by the Agence de Medicine Preventive (AMP), which deployed and supervised country and regional consultants to support influenza surveillance activities for a period of seven months, from May to December 2011 [9]. Among the eight countries selected for implementation of the SISA project, Sierra Leone was the only country that had no prior influenza surveillance activity in place. Thus, the challenge was not simply to strengthen existing surveillance, but to implement a functional ISS system in a country emerging from a decade-long civil war with a health system challenged by a lack of resources, infrastructure and trained work force.

The objectives of this paper are threefold:to describe the approach used in Sierra Leone in the development and implementation of a functional ISS system; to illustrate the challenges and solutions encountered in developing a surveillance system in a resource-poor country previously lacking an influenza monitoring system; and to provide resource-poor countries, external funders and implementers with a better understanding of the opportunities and challenges of developing new surveillance activities.

## Methods

The SISA project was implemented in Sierra Leone using a common methodological framework including development of initial situation assessment and recommendations for improvement; drafting of relevant country-specific surveillance protocols and standard operating procedures (SOPs); training of sentinel surveillance and data management staff; and supervision of surveillance activities [9]. The methods used in Sierra Leone were adapted to its specific context, using the Integrated Disease Surveillance and Response (IDSR) strategy as a platform to establishing the ISS system. The IDSR is a comprehensive regional framework for strengthening national public health surveillance and response systems in Africa, supported by WHO.

An initial briefing meeting at the start of the project brought together national program managers and other stakeholders concerned with surveillance activities. All relevant information concerning influenza, including the background, situation analysis, objective and proposed process for implementation of the ISS system in the country, was provided. This initial meeting was instrumental in identifying designated personnel to be involved in the assessment exercise and to ensure leadership at the national level. Throughout the course of implementation,particular attention was placed on training and regular supervision of sentinel surveillance staff. Monthly review meetings were conducted to closely monitor and support the initial phase of implementation of ISS activities.

The systematic approach followed in establishing a functional ISS system is described below.

### Assessment of surveillance functions and system analysis

**Figure 1:**
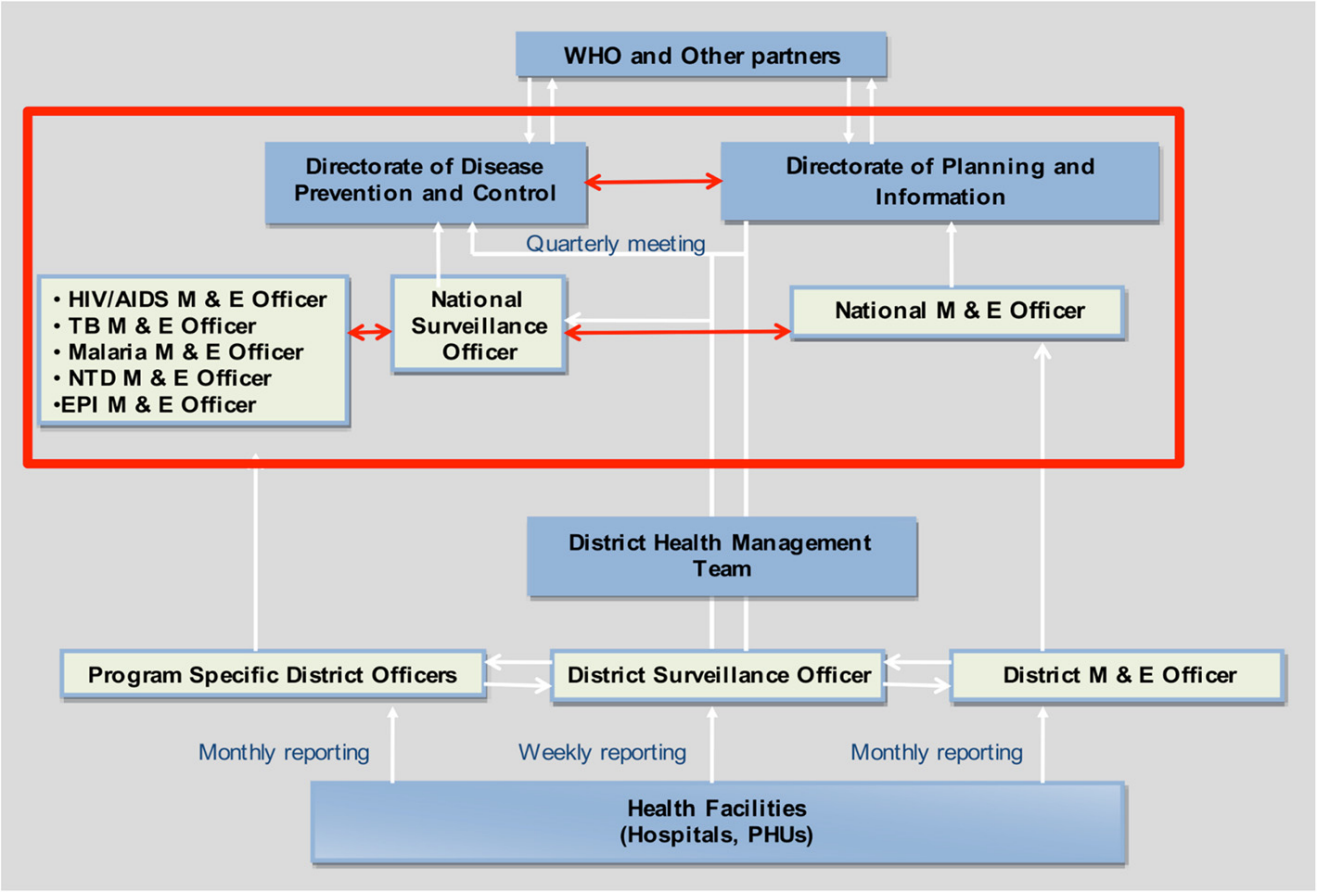
Surveillance information flow in Sierra Leone.

Initial activity focused on understanding the organization of surveillance function, including data management at various levels, laboratory support services and availability of suitable health facilities for integrating ISS into existing disease surveillance systems. The methods included the review of relevant documents, visits to the National Ministry of Health of Sierra Leone (MOHS), the Directorate of Disease Prevention and Control (DPC), the National Disease Surveillance Programme (NSD), the District Health Management Team (DHMT) of the Western area, the Central Public Health Reference Laboratory (CPHRL) and the health facilities in the district. Figure 1 illustrates the flow of surveillance information between and within levels of the health system in Sierra Leone, showing the routine flow of surveillance data from reporting sites (health facilities) to the regional and national level, and subsequently to the central health authorities.

Sierra Leone has implemented the IDSR strategy, coordinated by the National Surveillance Unit (NSU) within the DPC; IDSR promotes the integration of resources through the coordination and streamlining of common surveillance activities. By integrating multiple surveillance systems, limited personnel and scarce resources can be used more efficiently. In the IDSR implementation framework, epidemiologic surveillance is linked with laboratory support in order to produce relevant information for taking public health action [10]. Ultimately, IDSR is expected to lead to improved surveillance information flow within and between levels of the health system.

At the time of the assessment, it appeared that the IDSR infrastructure was an appropriate platform in which to integrate the ISS system to ensure sustainability. The sustained laboratory support from diverse partners including the WHO, CDC, Association of Public Health Laboratories (APHL), UNICEF and the Global Fund to Fight AIDS, Tuberculosis and Malaria (GFATM) had gathered momentum to upgrade the CPHRL laboratory capacity to Bio-safety Level 2 (BSL-2) standards. The laboratory was in the initial stages of conducting testing of bacterial meningitis samples for surveillance and HIV polymerase chain reaction (PCR) testing for the early infant diagnosis of HIV. The available PCR machines could ideally be used to identify influenza viruses. However, prior to initiation of influenza testing, building of laboratory human resource capacity and supply of primers would be required.

#### Conclusions and recommendations of initial assessment

Based on the findings of the assessment, it was feasible to establish an ISS system in Sierra Leone without delay. Consequently, health facilities for the ISS system were selected based on WHO criteria, which included administrative division, geographical location, public-private mixture, institutional capacity to carry out surveillance activities, and staff interest in participation [11]. Recommendations of the initial assessment included the need to collaborate with the Institut Pasteur in Dakar, Senegal, for influenza laboratory testing while strengthening the ongoing effort to build the infrastructure and human capacity to allow for in-country testing within the shortest time possible.

### Defining roles and responsibilities in the ISS system sites

Applying the WHO criteria, the following four health facilities in the Western area of Freetown were selected as initial sentinel sites for implementation of the ISS: Jenner Wright Children’s Clinic, Ola During Children’s Hospital, Lumley Government Hospital, and Blue Shield Hospital (private). Surveillance information flow within the health system, which included upwards transmission of data and reports, as well as communicating feedback to sentinel sites and stakeholders, was as follows:

#### Sentinel site staff

The responsibilities of sentinel site staff included collecting epidemiological and laboratory data, transmitting these data to the District Surveillance Officers (DSOs), and collecting, storing and delivering patient specimens to staff at CPHRL.

#### Central national public health reference laboratory

The molecular unit of the CPHRL served as the National Influenza Reference Unit (NIRU) and was responsible for developing protocols for influenza specimen chain of custody and transport, and for training of sentinel site laboratory personnel in specimen handling, bio-safety measures and cold chain maintenance. The CPHRL was also responsible for collecting samples from sentinel sites, logging information, packing and shipping samples to the Institut Pasteur in Dakar, Senegal, and entering laboratory data into the WHO FluNet database.

#### National surveillance unit (NSU), ministry of health and sanitation

The NSU was responsible for analyzing the epidemiological and laboratory data supervision and providing feedback to sentinel site staff and other stakeholders. The NSU was also responsible for entering epidemiological data into the global database FluID. The DSOs were in charge of reviewing and compiling weekly sentinel site data.

### Developing guidelines and tools

The Sierra Leone national influenza sentinel surveillance protocol and SOPs for influenza sample collection, sample handling, packaging, shipping and transport, and data handling and sharing were developed based on the generic WHO guidelines. Additional protocols were adopted for entering epidemiological and virological data into the global databases, FluID and FluNet, which are key tools in monitoring global influenza trends in real-time. Information from FluID and FluNet is available to health professionals and policy makers to assist in making informed decisions regarding influenza management. Using these databases, surveillance activity in Sierra Leone is incorporated into a global network for influenza epidemiological data collection.

#### Case definitions

Standardized case definitions were adopted to identify influenza-like illness (ILI) and severe acute respiratory infection (SARI) cases. An ILI case was defined as “a person with respiratory illness with a measured temperature greater than 38°C and an onset of a cough within the last seven days”. A SARI case was defined as “a person with respiratory illness with a history of or measured temperature greater than 38°C, a cough, and onset of shortness of breath or difficulty breathing that required hospitalization within the last seven days”.

### Training, supporting and supervising staff

The training workshop targeted all staff involved in surveillance, including surveillance officers at the national and district level, clinicians, laboratory technicians, monitoring and evaluation officers in charge of data at the selected health facilities, and staff at CPHRL. The topics covered in the training included an overview of influenza epidemiological patterns, clinical and virological diagnosis of influenza, and specimen collection, handling and transportation. Data management, including data collection, collation, analysis and flow, was also discussed. At the national level, additional information on weekly summary reporting and data analysis using EpiInfo software was discussed.

On-site support at sentinel sites covered practical demonstration of nasal swab sample collection, data recording, compilation of weekly summaries, and transfer of samples. Additional support included demonstration for correct sample handling, storage, and assurance of cold chain integrity and usage of sample custody forms.

The NSU conducted regular supervision to facilitate smooth operation of the implementation. The DSOs in charge of collecting and compiling weekly sentinel site data also provided additional support to sentinel sites, including cleaning data to maintain data quality and timeliness. Regular review meetings were held, bringing together all staff engaged in sentinel influenza surveillance from sentinel sites, CPHRL, MOHS and WHO. The objectives of the meeting were to determine the progress of implementation, identify challenges, and propose solutions and elaborate subsequent steps in the process of implementation.

### Influenza surveillance processes

The sentinel surveillance system formally began on August 19, 2011. The implementation was closely monitored and supported through onsite technical assistance, regular supervision and monthly review meetings.

#### Case recording and sample collection

A sampling method was used for ILI cases based on the patient load at health facilities and the capacity of the laboratory to process samples. Accordingly, a nasal swab sample from every fourth ILI patient was collected. For SARI, a nasal swab was collected from all cases. Prior to sample collection, an explanation of the procedure was provided and verbal consent was obtained from the patient or guardian. Nasal swabs were collected according to the procedures described in the SOPs. The following data were recorded for each patient sampled: sentinel site identification number, date of symptom onset, date of specimen collection, date of hospitalization (for SARI cases), sex, age, temperature, pregnancy status, seasonal influenza vaccine status, antiviral use, and any comorbidities.

#### Laboratory specimen handling

Specimens were stored at sentinel sites in appropriate viral transport media at 2-8°C until collection by the CPHRL. Sentinel sites notified the CPHRL regarding samples that were collected and ready for pick-up. Samples were picked up by the CPHRL within 48 hours of collection. At the CPHRL, the specimens were logged and packaged for shipment to the Institut Pasteur in Dakar, Senegal.

#### Laboratory testing

Reverse transcriptase PCR (RT-PCR) testing for influenza A and B was conducted at the Institut Pasteur in Dakar, Senegal. The results were communicated to the MOHS and also back to the sentinel sites from which the sample originated.

## Results and discussion

During the SISA project surveillance data collection period in Sierra Leone, from August to December 2011, 1,235 ILI cases (12.9% of the total consultations) and 282 SARI cases (4.6% of all hospitalizations) were identified in the four sentinel sites (Table 1). Nasal samples were collected from 268 ILI and 238 SARI cases with ages ranging from 1 month to 62 years. Laboratory results were available for 473 samples, of which 12.7% tested positive for influenza virus ribonucleic acid (RNA). Among the positive samples, 55 (91.7%) had subtype A(H3N2), 4 (6.7%) had subtype A(H1N1)pdm09 and 1 (1.7%) had a mixed infection of subtype A(H1N1)pdm09 and A(H3N2). No influenza B subtypes were detected. The majority (36%) of ILI and SARI cases were detected in children under the age of four.

**Table 1:** Number of influenza samples collected per sentinel site in Sierra Leone (August 2011 to December 2011)

**Sentinel site**	**Number of ILI cases**	**Number of SARI cases**	**Number of samples collected**
Jenner Wright Hospital	677	0	163
Ola During Hospital	0	249	205
Lumley Hospital	487	25	116
Blue Shield Hospital	71	8	22
**Total**	1,235	282	506

### Follow-up

The SISA project provided in-country technical support from May to December 2011. Following this, Sierra Leone continued influenza surveillance successfully with previously existing mechanisms for implementation of IDSR. From January to December 2012, a total of 1,363 ILI and 281 additional SARI cases were reported, of which 264 ILI and 201 SARI cases were sampled. Despite fewer samples being collected and a delay in reporting in comparison to the initial project period, the surveillance activity continued to provide valuable data for national and global databases.

**Figure 2:**
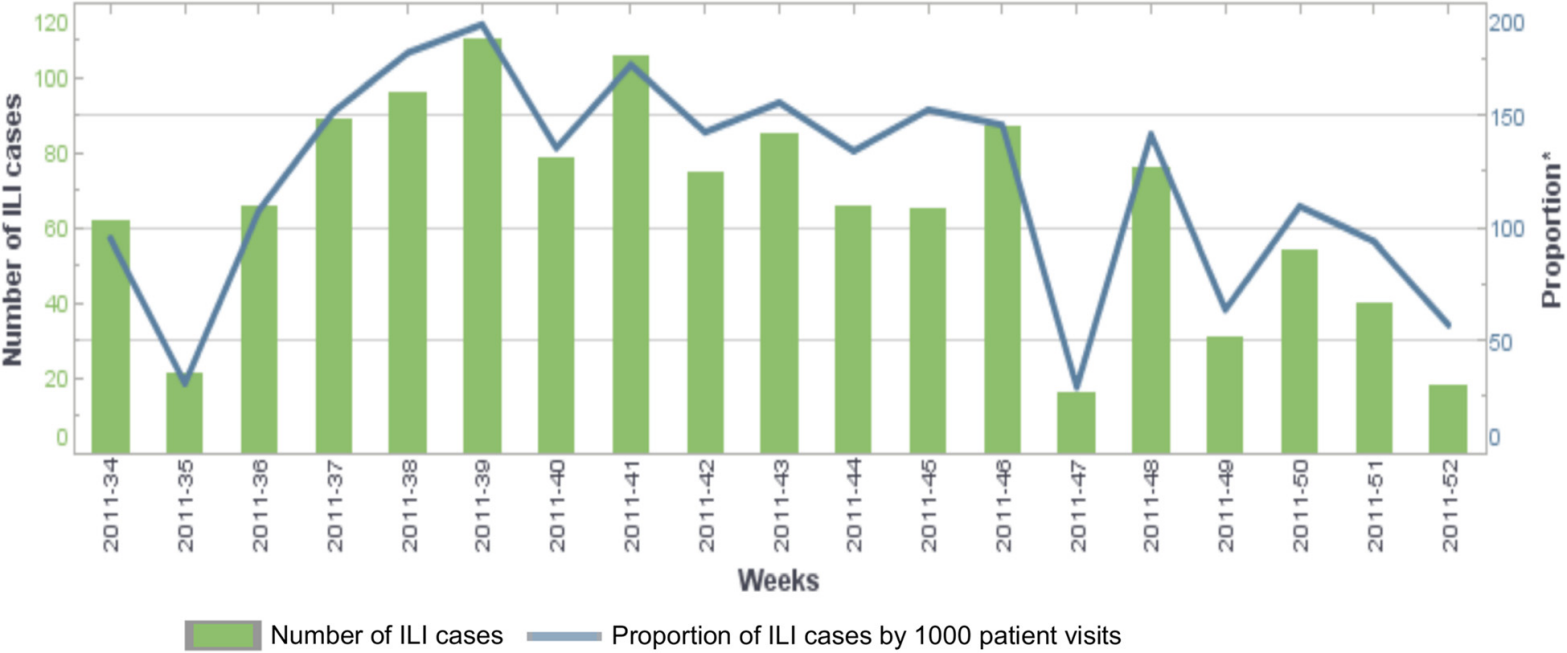
Number of influenza-like illness (ILI) cases identified and proportion of ILI cases by 1,000 patient visits in Sierra Leone (August 2011 – July 2012).

**Figure 3:**
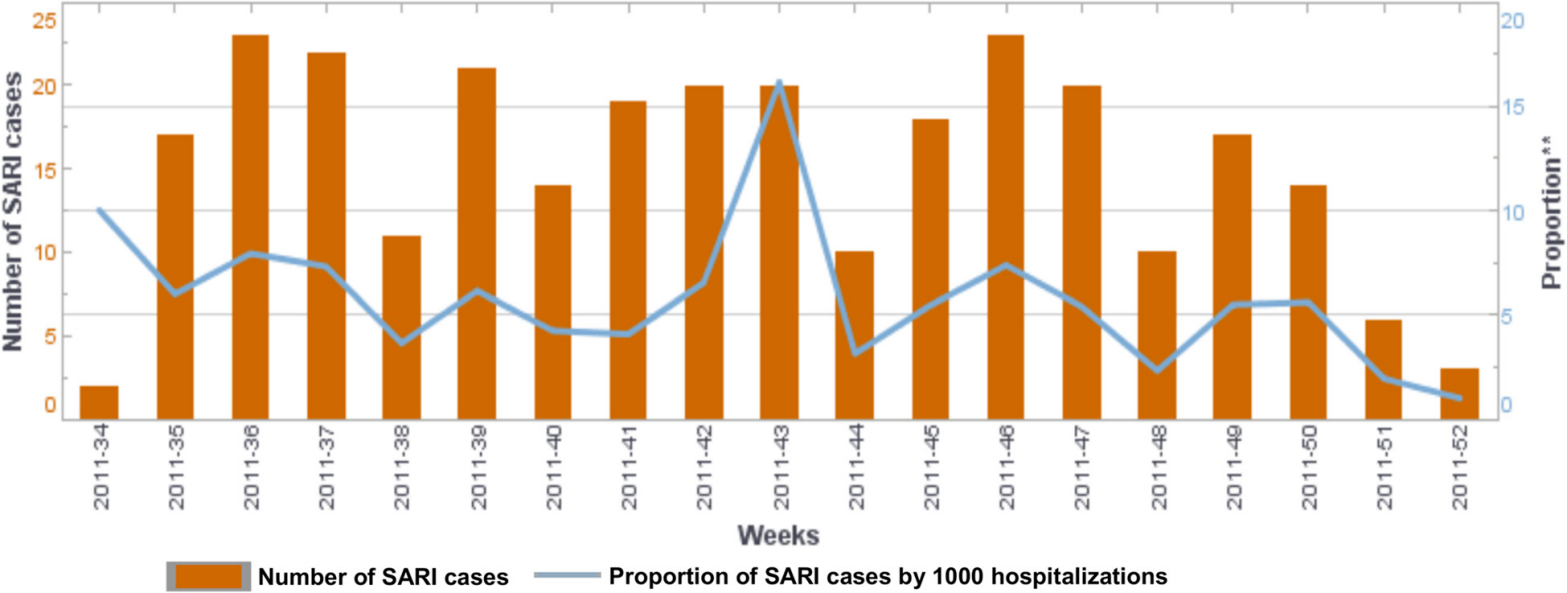
Number of severe acute respiratory infection (SARI) cases identified in Sierra Leone (August 2011 – July 2012).

**Figure 4:**
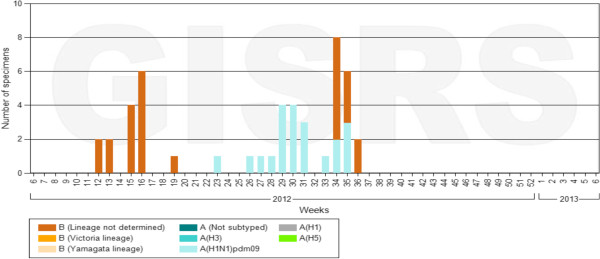
Number of specimens identified positive for influenza by subtype in Sierra Leone (August 2011 – July 2012)

By the end of December 2012, a total of 2,598 ILI and 570 SARI cases were reported. Figures 2 and 3 show the weekly number of ILI and SARI cases identified as well as the proportion of ILI and SARI cases among outpatient and inpatient visits. During this period, a total of 582 ILI and 532 SARI cases were sampled. Laboratory results were available for 942 samples, of which 108 (11.5%) tested positive for influenza. Among the positive samples, 55 (50.9%) had subtype A(H3N2), 26 (24.1%) had subtype A(H1N1)pdm09, 26 (24.1%) had influenza B, and 1 (0.93%) had a mixed infection of subtype A(H1N1)pdm09 and A(H3N2) (Figure 4).

### Challenges and solutions

The major challenges and constraints in the implementation of a successful ISS in Sierra Leone included rapid turnover of sentinel site staff; weak supply chain management leading to shortage of supplies, in particular sample collection kits; inadequate supply of fuel for transportation of samples from collection site to the CPHRL; and a lack of continuous electrical power supply in the health facilities to maintain cold chain integrity. Additional constraints included delays in uploading data to the global databases and prolonged turnaround time for laboratory results.

The challenges highlighted the critical need for proper situation analysis, adequate preparation, prepositioning necessary materials and supplies, and prior anticipation of potential risks to ensure a smooth implementation process. Many of the challenges and constraints were addressed in the course of project implementation through consultations facilitated by the external consultant to establish solutions as they are identified. The constraints were usually identified during follow-up visits, review meetings or when reported by the concerned sites. Solutions included working with the health facility to assign new staff to be trained, increasing the frequency of visits to the site with new staff, and putting systems in place to ensure regularity and predictability of essential supplies including fuel for transportation of specimens. In some instances, alternative solutions could be used to overcome some of the challenges. For example, in health facilities without dependable power to maintain cold chain, frozen ice packs were provided and the frequency of transportation of samples was increased.

### Lessons learned

The successful implementation of an ISS system in Sierra Leone was the result of many factors, including a systematic approach, national ownership, appropriate timing, and external support. More specifically, it was achieved through:

•Focused advisory and technical support with a timeline for achieving specific targets

•Assessment of the capacity of current surveillance systems and opportunities to build upon existing framework

•Establishment of a systematic approach with clearly defined roles

•Appropriate timing to build upon opportunities for integration or synergy with existing programs

•Promotion of national ownership through early engagement and regular update of decision makers

•Ensuring stable funding sources to cover core functions (review meeting costs, etc.)

•Securing additional funding to cover specimen collection, laboratory costs, consumables, etc.

•Close monitoring of progress with update and feedback at regular review meetings

•Motivation of staff through recognition and other non-monetary incentives

### Systematic approach

The project was implemented using a deliberate systematic approach, beginning with a thorough assessment of Sierra Leone’s existing surveillance system capabilities. The national IDSR network was used as a platform to integrate the ISS and demonstrated that building on an existing framework was the most appropriate approach.

Training, which began after all stakeholders were identified and committed to be part of the sentinel site, occurred at multiple levels and ensured that all involved with surveillance activities were properly trained before progressing to the implementation phase. A similar approach was used in data collection. The roles, responsibilities and reporting timelines were clearly defined on the surveillance data flow for each level to avoid confusion and to ensure regular submission of data. Using this approach ensured that the project was fully prepared to successfully implement the sentinel site surveillance.

### National ownership and motivation of surveillance staff

Another important factor for the success of the SISA project was the ownership and active leadership of the government of Sierra Leone. The ISS system implementation began with a briefing at the national level to ensure the participation of decision makers and staff involved in surveillance activities at various levels. Following the initial assessment, a consensus-building meeting was held where important information was communicated and the national action plan was developed using a participatory approach. The monthly review meetings that brought together focal persons from the various sentinel sites, CPHRL, and surveillance officers from the district and national levels served for sharing experiences, providing feedback and troubleshooting problems. Allowing staff members to directly participate in the evaluation and management of the surveillance activities and openly recognizing their work, increased staff motivation and dedication.

### Appropriate timing

The timing of the project coincided with the strategic planning for IDSR, which allowed for its integration with the overall surveillance function and provided additional support for the activity. Most importantly, the project benefited from the national leadership, committed partners, team approach and continuous engagement and motivation of all staff involved. The introduction of the SISA project also coincided with the ongoing efforts of partners, such as WHO and CDC, in building the capacity of the CPHRL. Additionally, partnerships such as the Institut Pasteur in Dakar, Senegal, and the Ghana National Influenza Center were beneficial in providing laboratory services unavailable in-country.

### External support

The focused technical and financial support through the SISA project was critical for the success of establishing the system for ISS in Sierra Leone. However, continuation of project support following the initial phase could have allowed for better consolidation of the achieved results and detailed analysis, including seasonal variation, of the information from the epidemiological and virological data in the country. Despite the above limitation of the project, the integration of the ISS into the IDSR is an effective and efficient approach that will allow for sustainability. These efforts will require the continued support of partners such as WHO and CDC in order for Sierra Leone to maintain generation of influenza surveillance data that is critical for disease control efforts at local and international levels.

## Conclusions

The WHO SISA project demonstrated the feasibility of building a functional influenza surveillance system in Sierra Leone, integrated into the existing national IDSR system and generating relevant surveillance data within a seven-month in-country project phase and sustained with support of local partners.

Although the collaboration with influenza laboratories in the region was a useful initial step, the reliance on an out-of-country laboratory for testing required additional organization, time, and increased funds. Strengthening and accreditation of the CPHRL of Sierra Leone will enable the national ISS system to function more efficiently and independently. Future laboratory related work should ensure a system for efficient specimen handling, regular supply of markers, and participation in quality assurance to ensure that the CPHRL is advanced to the list of National Influenza Center status.

Despite the challenges and limitations, the ISS system in Sierra Leone produced valuable results that were incorporated into the global databases FluID and FluNet. Additionally, one year following cessation of active external technical support by SISA, the system continues to be operational and effective in monitoring the burden of influenza. National ownership was demonstrated to be a critical element in the success of the project and is critical for sustainability of the ISS system and other future surveillance activities. The ISS system, if sustained long-term, would provide valuable data to determine epidemiological and virological patterns and trends to assess the influenza disease burden that will ultimately guide national control strategies.

## Abbreviations

APHL: Association of Public Health Laboratories; CDC: US Centers for Disease Control and Prevention; CRHRL: Central Public Health Reference Laboratory; DHMT: District Health Management Team; DPC: Directorate of disease prevention and control; DSO: District surveillance officers; IDSR: Integrated disease surveillance and response; ISS: Influenza sentinel surveillance; MOHS: Ministry of health and sanitation; NSD: National disease surveillance program; RT-PCR: Reverse transcriptase - polymerase chain reaction; SISA: Strengthening influenza sentinel surveillance in Africa project; SOPs: Standard operating procedures; WHO: World Health Organization.

## Competing interests

CA Steffen works for the Agence de Médecine Préventive (AMP), which receives unrestricted support from Sanofi-Pasteur and grant-specific support from Crucell, GlaxoSmithKline, Merck, Novartis Vaccines, Pfizer and Sanofi Pasteur. All other authors have declared that no competing interests exist.

## Authors’ contributions

SK and IC conceived the study. CA was the overall SISA project coordinator for AMP and provided scientific advice and technical input to the manuscript as well as revision work. SK, IC, CA, IW, and FD organized and coordinated collection of field data. SK, IC, and FK analyzed data. SK drafted the manuscript. SK, CA, WA, and KV finalized the manuscript. AJ, AY, FK provided feedback to the manuscript. All authors read and approved the final manuscript.
